# Endothelial Microvesicles and Soluble Markers of Endothelial Injury in Critically Ill Newborns

**DOI:** 10.1155/2018/1975056

**Published:** 2018-07-19

**Authors:** Veronika Vítková, Martin Pánek, Petr Janec, Michaela Šibíková, Václav Vobruba, Martin Haluzík, Jan Živný, Jan Janota

**Affiliations:** ^1^Department of Neonatology, Thomayer Hospital Prague, Videnska 800, 14059 Praha 4, Czech Republic; ^2^Institute of Pathological Physiology, First Faculty of Medicine, Charles University, U Nemocnice 5, 12853 Praha 2, Czech Republic; ^3^Department of Neonatology, Masaryk Hospital Usti nad Labem, Krajska zdravotni, Socialni pece 3316/12A, 40113 Usti nad Labem, Czech Republic; ^4^Third Faculty of Medicine, Charles University, Ruska 87, 10000 Praha 10, Czech Republic; ^5^Department of Pediatrics, General University Hospital in Prague and First Faculty of Medicine, Charles University, Ke Karlovu 2, 121 00 Praha 2, Czech Republic; ^6^The Institute of Medical Biochemistry and Laboratory Diagnostics, First Faculty of Medicine, Charles University and General University Hospital, Kateřinská 32, 12853 Praha 2, Czech Republic

## Abstract

Neonatal systemic inflammatory response and multiple organ dysfunction syndrome are the main postnatal insults influencing mortality and morbidity. Critically ill newborns with high predicted mortality are supported by extracorporeal membrane oxygenation (ECMO). Biomarkers of inflammatory response and endothelial injury can be used for early diagnosis and treatment of critical neonatal situations. The aim of our study was to explore plasma proteins and endothelial microvesicles as markers of inflammation and endothelial activation in newborns on ECMO and to compare them with healthy neonates. Thirteen newborns on ECMO and 13 healthy newborns were included in the study. Plasma soluble biomarkers were measured using multiplex immunoassay based on Luminex® xMAP multianalyte profiling platform. The total microvesicle count and plasma level of surface antigen-specific microvesicles were determined by flow cytometry. The plasma concentration of cell-derived microvesicles was measured using annexin-V labeling, and the endothelial origin of microvesicles was determined using lineage-specific antigen labeling of endothelial cell/microvesicle markers (endoglin/CD105, PECAM1/CD31, VEGFR2/CD309, and MadCAM1). Inflammatory markers (procalcitonin, IL-1*β*, IL-6, and IL-22) were increased in the ECMO group (*P* < 0.01). The assessment of endothelial markers showed higher concentrations of endocan and angiopoietin-2 (*P* < 0.01) in the ECMO group while VEGF in the ECMO group was significantly lower (*P* < 0.01). In the ECMO group, the concentration of annexin-V-positive microvesicles (total microvesicles) and endothelial microvesicles expressing mucosal vascular addressin cell adhesion molecule 1 (MadCAM1) was increased (*P* = 0.05). In summary, we found increased concentrations of soluble inflammatory and endothelial markers in the plasma of critically ill newborns with multiple organ dysfunction. Increased plasma concentrations of microvesicles may reflect the activation or damage of blood cells and vasculature including endothelial cells. The measurement of cell membrane-derived microvesicles may be added to the panel of established inflammatory markers in order to increase the sensitivity and specificity of the diagnostic process in critically ill newborns.

## 1. Introduction

Neonatal mortality and morbidity are important factors reflecting the level of medical care [1]. Pathological situations and diseases of mother and newborn during perinatal period influence significantly neonatal mortality and long-term morbidity [2]. Neonatal pathologies causing multiple organ dysfunction—prematurity, systemic infections, and perinatal asphyxia—are the main postnatal insults influencing neonatal mortality and morbidity [2, 3]. Critically ill newborns with high predicted mortality and maximum conventional support of vital functions are candidates for extracorporeal membrane oxygenation (ECMO)—an invasive method of life support which improves their survival [4, 5]. These patients have clinical and laboratory signs of systemic inflammatory response and multiple organ dysfunction or failure [5, 6]. The search for novel markers helping the early diagnosis, assessment of severity, and early treatment of serious perinatal complications associated with systemic inflammation and organ dysfunction is the priority of current neonatology [3].

Neonatal mortality, morbidity, and the successful approach to therapy of critically ill newborns depend on the understanding of the pathophysiology of defensive inflammatory response to perinatal insults. Biomarkers and mediators of inflammatory response can be used for early diagnosis and treatment of critical neonatal situations associated with multiple organ dysfunction [7].

The endothelium plays an important role in many physiological functions, including the control of vasomotor tone, blood cell trafficking, hemostatic balance, permeability, proliferation, survival, and innate and adaptive immunity. The endothelium is involved in most disease states, either as a primary determinant of pathophysiology or as a victim of collateral damage [8, 9]. Almost every stimulus leading to a systemic inflammatory response, that is, severe infection, trauma, excessive tissue breakdown, solid tumors, leukemia, pregnancy-associated complications, vascular anomalies, liver failure, and toxicological or immunological responses, and activation of the coagulation system can be associated with endothelial damage. The level of systemic endothelial injury limits the reversibility of organ dysfunction. Endothelial cell release during stimulation, activation, and injury soluble biomarkers. Biomarkers can be detected and quantified in the peripheral blood [9]. Detection of biomarkers of endothelial injury in peripheral blood helps to detect and quantify the severity of systemic, cardiovascular, and infectious diseases in adults, children, and newborns [10–12].

Somatic cells, including endothelial cells, secrete a variety of small, defined cell membrane-derived vesicles termed exosomes, microvesicles, and apoptotic bodies. Cell membrane microvesicles (microparticles) are currently judged as important messenger and indicators of cell activation, apoptosis, or tissue degeneration [13, 14]. Microvesicles (~100–1000 nm) are released by budding from the plasma membrane of most eukaryotic cells undergoing activation and are characterized by exposure of membrane proteins and phosphatidylserine residues on their outer surface. Elevated levels of circulating microvesicles are associated with various vascular pathologies, inflammatory, malignant, and hematologic disorders [15].

Changes in the levels of endothelial microvesicles were detected, measured, and might be potentially used to evaluate the severity of the disease in serious systemic and cardiovascular pathologies in adults [16]. In neonates and children, endothelial microvesicles were determined in patients with vasculitis and blood group incompatibility [17, 18].

Maternal and neonatal biomarkers of endothelial injury in diseases associated with perinatal period can be used as potential diagnostic markers influencing early detection and treatment of diseases with negative impact on neonatal mortality and morbidity. This may lead to decrease in neonatal mortality and long-term morbidity. There is limited information regarding endothelial cells, endothelial dysfunction, and endothelial damage in newborns. The complex assessment of maternal and neonatal markers produced by endothelium (vasoactive substances, cytokines, adhesive molecules, growth, and angiogenic factors) during perinatal period was not done yet. There is currently no information available regarding endothelial microvesicles in critically ill newborns.

The aim of our study was to explore biomarkers of endothelial activation and damage in critically ill newborns during extracorporeal membrane oxygenation and to compare them with biomarkers in healthy term neonates.

## 2. Materials and Methods

### 2.1. Patients

Thirteen patients on extracorporeal membrane oxygenation (ECMO group) admitted to ECMO Center, General University Hospital in Prague from October 2012 to April 2016 were enrolled in the study after informed consent of their parents had been obtained. Thirteen healthy term newborns (control group), admitted to Department of Neonatology, Thomayer Hospital Prague, Czech Republic, from January 2016 to July 2016 were enrolled in the study after informed consent of their parents had been obtained. The study was approved by institutional ethics committee in both participating hospitals.

### 2.2. Blood Sample Processing and Storage

Blood samples for both groups of patients were collected within the first week of life into sodium citrate. In the ECMO group, blood samples were collected during ECMO support, and in the control group, samples were collected when bloods for mandatory metabolic screening were taken. Blood samples were processed by standardized method published previously and stored at −80°C [15]. Samples were processed within 60 minutes of collection. Full blood was centrifuged at the 2700*g* for 15 min at 10°C followed by the second spin of collected plasma at 2700*g* for 10 minutes at 10°C. Collected plasma was then aliquoted into screw cap tubes and stored at −80°C until used for isolation of microvesicles or measurement of soluble markers.

### 2.3. Soluble Biomarker Measurement

Soluble biomarkers were measured using multiplex magnetic bead immunoassay based on Luminex® xMAP multianalyte profiling platform and analyzed on MAGPIX® System (Merck Millipore) at the Institute of Medical Biochemistry and Laboratory Diagnostics of The General University Hospital and of the First Faculty of Medicine of Charles University in Prague. The following premixed magnetic bead kits were used for plasma sample analysis: LXSAHM-07: endoglin (BR22), IL-1*β* (BR28), IL-22 (BR35), IL-6 (BR13), procalcitonin (BR39), VCAM-1 (BR57), and VEGF-A (BR26) and LXSAHM-08: angiopoietin-2 (BR26), endothelin-1 (BR76), ICAM-1 (BR61), IL-1*α* (BR38), IL-17A (BR42), IL-18 (BR78), IL12 p70 (BR563), TNF-*α* (BR12) (both from Biotechne R&D Systems), and Milliplex MAP human soluble receptor magnetic bead panel (HSCRMAG-32K-02: VEGFR1 and VEGFR2) and cardiovascular disease (CVD) magnetic bead panel (HCVD1MAG-67K-01: endocan1/ESM1) (Merck).

### 2.4. Isolation and Measurement of Microvesicles

**Figure 1:**
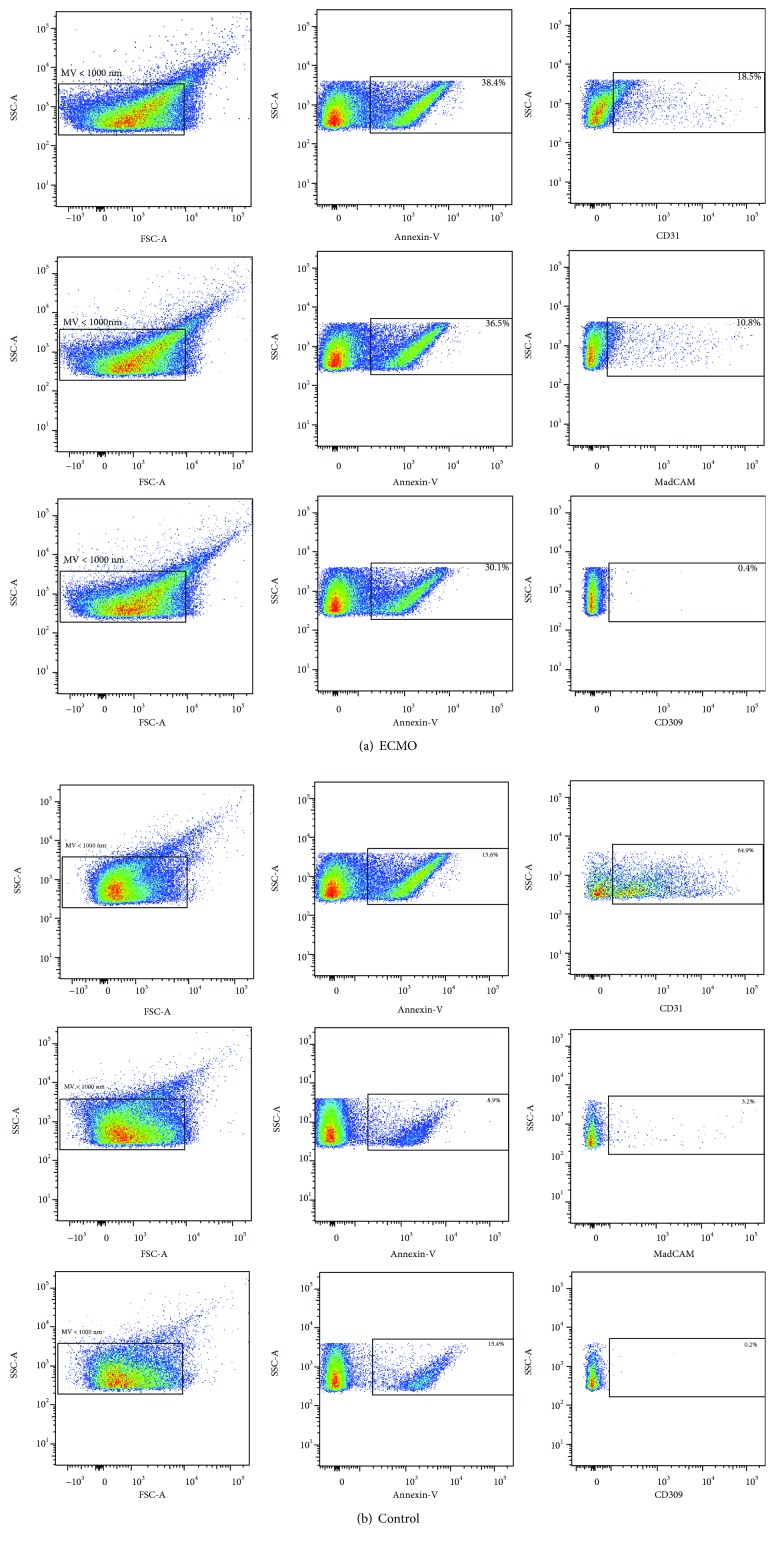
Representative dot plots showing gating strategy for the analysis of antigen-specific microvesicles: (a) ECMO patient; (b) control. Based on the size analysis of the standard ApogeeMix beads, the gate for microvesicles (MV < 1000 nm) was created (left column). From the gate MV < 1000 nm, the phosphatidyl serine-positive microvesicles were identified based on the labeling with annexin-V and the gate of MV < 1000 nm/annexin-V+ was created (middle column). The annexin-V-positive microvesicles were then analyzed for the positivity of individual cell-specific markers PECAM/CD31, MadCAM1, and VEGFR2/CD309 (right column).

Plasma cell membrane-derived microvesicles were isolated by centrifugation of stored platelet-free sample aliquots at 21000*g* for 10 min. The total microvesicle count and the number of surface antigen-specific microvesicles were determined by flow cytometry (BD FACS CantoII). The plasma microvesicles were identified based on size discrimination and annexin-V FITC (Apronex Biotechnologies) labeling. Cell origin of microvesicles was determined using lineage-specific antigen labeling with CD105 AF647 (BD Biosciences), platelet and endothelial cell adhesion molecule 1 (PECAM1)/CD31 BV510 (BD Biosciences), VEGFR2/CD309 AF647 (BD Biosciences), and mucosal vascular addressin cell adhesion molecule 1/MadCAM APC (Biotechne R&D Systems). A representative dot plots describing the gating strategy to identify the vesicles in the flow cytometry data are shown in Figure 1.

### 2.5. Flow Cytometry Setup

The forward (FSC) and side scatter (SSC) were adjusted using polystyrene latex and silica bead mixture of defined sizes (ApogeeMix, Apogee). ApogeeMix contains aqueous mixture of plastic spheres with diameters 180 nm, 240 nm, 300 nm, 590 nm, 880 nm, and 1300 nm with refractive index *ɳ* = 1.43 (silica), and 110 nm and 500 nm green fluorescent beads with refractive index *ɳ* = 1.59 (latex). We were able to discriminate bead size based on FSC and SSC between 300 nm and 1000 nm. Data analysis was performed from 25,000 counted events using BD FACSDiva Acquisition and analysis software. The results were expressed as the number of microvesicles per microliter (MV/*μ*l) of plasma calculated based on the input volume of plasma and cytometer flow rate.

### 2.6. Statistical Analysis

GraphPad Prism5 system was used for data analysis. The Mann–Whitney *U* test was performed to determine the statistical significance between the groups. All statistical tests are based on a significance level of *P* ≤ 0.05.

## 3. Results

Thirteen newborns on extracorporeal membrane oxygenation (ECMO group) and 13 healthy term newborns (Control group) were included in the study. Characteristics of ECMO and control groups are shown in Table 1. There were no significant differences in gestational age, birthweight, and gender between groups. Characteristics of the ECMO group were as follows: veno-arterial support in 11 patients (85%) and veno-venous support in 2 patients (15%). Primary diagnosis was pulmonary hypertension of the newborn in 7 patients (55%), sepsis in 2 patients (15%), congenital diaphragmatic hernia in 2 patients (15%), and meconium aspiration in 2 patients (15%). One patient died during ECMO, and 2 patients died in hospital after ECMO support.

Plasma concentrations of soluble inflammatory and endothelial markers are shown in Tables 2 and 3. Inflammatory markers were significantly increased in the ECMO group, increase in TNF*α* did not reach statistical significance (*P* = 0.0616). The assessment of endothelial markers showed significantly higher concentrations of endocan (*P* = 0.0005) and angiopoietin-2 (*P* = 0.0013) in the ECMO group and significantly higher concentrations of VEGF in the control group (*P* = 0.0001).

**Figure 2:**
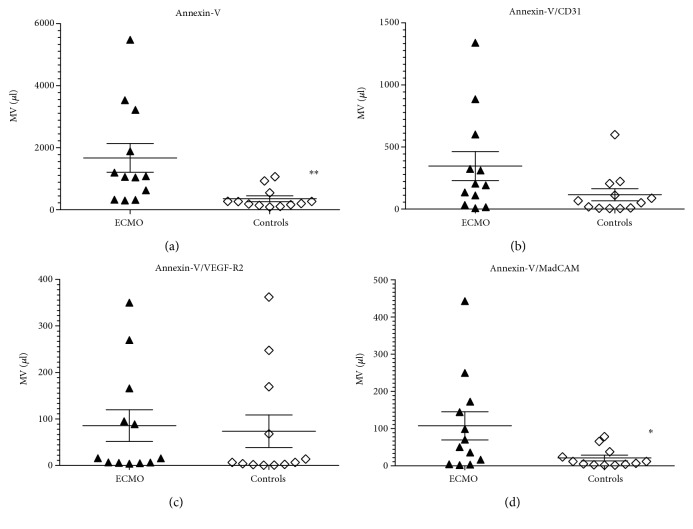
Concentrations of microvesicles (MV) in the plasma of newborns on extracorporeal membrane oxygenation group (ECMO) and term newborn group (controls): (a) annexin-V-positive MV; (b) annexin-V, PECAM/CD 31-positive MV; (c) annexin-V, VEGFR2-positive MV; (d) annexin-V, MadCAM1-positive MV. Data presented as values of individual patients and mean (*n* = 12 for each group). The error bars represent the standard error of the mean (SEM) (^∗^*P* ≤ 0.05, ^∗∗^*P* ≤ 0.01).

Plasma concentrations of microvesicles are shown in Figure 2. Concentration of annexin-V-positive microvesicles (total microvesicles) was significantly increased in the ECMO group (*P* = 0.0007). From endothelium-specific microvesicles, the only significantly increased were those expressed mucosal vascular addressin cell adhesion molecule 1 (MadCAM1) (*P* = 0.05). Other endothelium-specific microvesicles (PECAM/CD31 positive, VEGFR2 positive) were increased in the ECMO group, but did not reach statistical significance (*P* = 0.0606 and *P* = 0.2986, resp.).

## 4. Discussion

Neonatal extracorporeal membrane oxygenation is an invasive method used for life support of critically ill newborns with cardiorespiratory failure and high predicted mortality. We analyzed levels of inflammatory markers and markers of endothelial impairment of newborns supported by extracorporeal circulation and compared them to healthy newborns. Both groups of patients showed significant differences in Apgar scores, which reflect expected problems of postnatal adaptation in the ECMO group. Control group samples were collected during mandatory metabolic screening tests as approved and allowed by Ethics committee, which should be between 48 and 72 hours of life. The blood samples of the ECMO group were collected significantly later, between 36 and 350 hours of life, after fulfilling the ECMO criteria, transport to ECMO center, initiation of ECMO, and obtaining the informed consent.

Soluble markers of inflammation were significantly increased in the ECMO group. This is an expected increase because of the presence of systemic inflammation and multiple organ dysfunction in all ECMO patients, and this finding is in agreement with published data [19, 20]. When assessing the data of ECMO patients who died (*n* = 3), we did not find any significant differences within measured parameters compared to the patients who survived.

Soluble plasma endocan and angiopoietin-2 were significantly increased in the ECMO group showing potential endothelial impairment in newborns with systemic inflammatory response and multiple organ dysfunction. Increased concentrations of endocan and angiopoietin-2 were associated with clinical worsening and progression into specific organ dysfunctions in adult septic patients and as predictors of multiple organ failure. Endocan was used as sensitive marker of systemic inflammation in neonatal sepsis. Our findings are in agreement with these adult and pediatric clinical data [21–23]. The lower VEGF concentration in the ECMO group might possibly reflect impaired normal increase described during early neonatal period in healthy newborns. VEGF is a paracrine regulator of endothelial cell differentiation, angiogenesis, and maintenance of existing vessels [24, 25]. Indications for ECMO in our group of newborns were severe pulmonary or cardiopulmonary compromise. Under these circumstances, the tissues may be incapable of responding to the hypoxia and inflammatory stimuli with an increase in VEGF production [26, 27]. Lower VEGF may contribute to the pathophysiology of acute lung injury and multiple organ dysfunction [28, 29].

Cell-membrane-derived microvesicle concentrations were higher in the ECMO group suggesting generalized endothelial and blood cell injury. We detected statistically significant increase in the concentration of mucosal endothelium marker MadCAM1 microvesicles. Systemic inflammation and multiple organ dysfunction in critically ill newborns are accompanied with circulatory failure. Redistribution of the circulation leads to underperfusion of the mucosa mainly in the gut [30, 31]. Mucosal perfusion impairment and reperfusion injury following the stabilization of systemic circulation due to extracorporeal circulation therapy might cause the injury of the mucosal vessel endothelium. These processes may possibly lead to the release of mucosal endothelial microvesicles recorded in our study. Recently, reports on ECMO model on newborn piglets described mechanisms of gut barrier dysfunction associated with extracorporeal membrane oxygenation. This would suggest that not only the health conditions but also the extracorporeal circulation itself may contribute to the mucosal endothelial injury followed by the release of mucosal endothelium microvesicles (MadCAM1+) [32, 33].

Other endothelium-specific microvesicles (PECAM/CD31 positive, VEGFR2 positive) were also increased in the ECMO group, but did not reach statistical significance. This may reflect the reaction of endothelial cells, probably expressed less in other organ systems than in the mucosa [14]. We can only speculate about reasons of MV release in the study group as there are currently no data available regarding endothelial MV release in critically ill newborns.

## 5. Conclusions

We found increased concentrations of soluble inflammatory and endothelial markers in the plasma of critically ill newborns with multiple organ dysfunction and systemic inflammatory response syndrome. Increased plasma concentrations of microvesicles may reflect the activation or damage of endothelial cells. The measurement of cell-membrane-derived microvesicles may be added to the panel of established inflammatory markers in order to increase the sensitivity and specificity of the diagnostic process in critically ill newborns. This is so far the first study describing the concentrations of endothelial microvesicles together with inflammatory and endothelial markers in critically ill newborns.

## Figures and Tables

**Table 1:** Characteristics of the ECMO and control groups.

Patients	ECMO group	Control group
Number	13	13
Birth weight (grams)	3377 (111)	3392 (125)
Gestational age (weeks)	38.9 (0.4)	39.4 (0.3)
Male	7 (54%)	7 (54%)
Apgar at 1minute	4.5 (0.9)^∗∗^	9.0 (0.5)^∗∗^
Apgar at 5 minutes	6.2 (0.8)^∗∗^	9.5 (0.3)^∗∗^
Apgar at 10 minutes	7.3 (0.6)^∗∗^	9.9 (0.2)^∗∗^
Blood collection (hours)	104 (23)^∗^	58 (2)^∗^

Data presented as mean (SEM) or number (%); ^∗^*P* ≤ 0.05, ^∗∗^*P* ≤ 0.01.

**Table 2:** Plasma concentrations of soluble inflammatory markers in the ECMO and control groups.

Inflammatory markers	ECMO group (pg/ml)	Control group (pg/ml)
TNF-*α*	12.53 (6.34)	3.26 (0.97)
Procalcitonin	16,220 (3749)^∗∗^	680 (260)^∗∗^
IL-1*β*	9.98 (2.53)^∗∗^	1.67 (0.31)^∗∗^
IL-6	2043.00 (1330)^∗∗^	7.33 (0.82)^∗∗^
IL-22	17.46 (4.73)^∗∗^	3.41 (1.37)^∗∗^

Data presented as mean (SEM); ^∗^*P* ≤ 0.05, ^∗∗^*P* ≤ 0.01.

**Table 3:** Plasma concentrations of soluble endothelial markers in the ECMO and control groups.

Endothelial markers	ECMO group (ng/ml)	Control group (ng/ml)
Endocan	2789 (271)^∗∗^	1496 (128)^∗∗^
Angiopoietin-2	21.44 (6.26)^∗∗^	6.93 (0.75)^∗∗^
VEGF	4.19 (1.17) × 0.10^−3^^∗∗^	88.50 (21.19) × 0.10^−3^^∗∗^
sVEGFR1	5866 (1354)	4330 (541)
sVEGFR2	6543 (979)	7398 (397)
Endothelin-1	6.63 (1.18) × 0.10^−3^	5.49 (1.90) × 0.10^−3^
VCAM-1	5580 (1158)	3193 (172)
ICAM-1	512 (81)	372 (175)

Data presented as mean (SEM); ^∗^*P* ≤ 0.05, ^∗∗^*P* ≤ 0.01.

## Data Availability

The data used to support the findings of this study are available from the corresponding author upon request.
